# Study of Serum Haptoglobin Level and its Relation to Erythropoietic Activity in Beta Thalassemia Children

**DOI:** 10.4084/MJHID.2015.019

**Published:** 2015-02-15

**Authors:** Seham M. Ragab, Manal A. Safan, Eman A. Badr

**Affiliations:** 1 Departments of Pediatrics Faculty of Medicine, Menoufia University. Egypt; 2 Medical Biochemistry Faculty of Medicine, Menoufia University. Egypt

## Abstract

**Background:**

Serum haptoglobin (Hp) is a reliable marker for hemolysis regardless the inflammatory state.

**Objective:**

We investigated the possible relation between Hp depletion and hemolysis severity, hepatitis C virus (HCV) infection and iron load in β-thalassemia children.

**Methods:**

Twenty two β-thalassemia major (TM),20 β-thalassemia intermedia (TI) children with 20 age and sex matched healthy controls were involved. Pre-transfusion hemoglobin level was considered. Serum ferritin, Hp and transferrin receptor levels (sTfR) (by ELISA ), alanine aminotransferase (ALT) and aspartate aminotransferase (AST) (by colorimetric method) were assayed. Markers of hepatitis C virus (HCV) were done by PCR.

**Results:**

The mean Hp levels among the studied groups were as follows; 8.02 ± 0.93 (mg/dl), 8.6 ±0.72 (mg/dl) and 122 ± 18.5(mg/dl) for TM, TI and the controls respectively. Both patient groups had significantly lower Hp level compared to the controls (P<0.0001) with significant lower level in TM compared to TI children ( P= 0.034). Significant inverse correlations were found between serum Hp and sTfR levels ( reflecting the erythropoietic activity) in thalassemia children combined and in each group (TM and TI) as well as among HCV infected children. STfR was the only significant independent predictor for serum Hp level (t= −5.585, P<0.0001). Among HCV infected patients, no significant correlation was found between serum Hp and serum transaminases.

**Conclusion:**

Serum Hp depletion in thalassemia had significant relation to disease severity and correlated well with their erythropoietic activity, as assessed by the measurement of sTfR without significant relation to HCV infection. Extensive multicenter studies are recommended.

## Introduction

Thalassemia syndromes are a heterogeneous group of inherited anemias characterized by defective synthesis of one or more of the globin chain subunits of the hemoglobin tetramer.^1^

Thalassemias are the commonest monogenic disorders in the world.^2^

β-thalassemia constitutes a major health problem in Egypt with an estimated carrier rate of 9–10%.^3^ It is an autosomal recessive disorder of hemoglobin synthesis caused by a direct down-regulation in the synthesis of structurally normal β chains. Due to the excess of α-globin chains relative to β-globin chains; α-globin tetramers (α4) are formed and interact with the red cell membrane, leading to hemolytic anemia and increased erythroid production.^4^

In the absence of stoichiometric production of α- and β-globin chains, and in the presence of elevated erythropoietin (EPO) levels, erythroid precursors continue to proliferate.^5^ Relative excess of α-globin synthesis leads to formation of hemichromes and increased erythroid precursor apoptosis causing ineffective erythropoiesis (IE)^4^ which is characterized by expansion, limited differentiation, and premature death of erythroid precursors before they reach the reticulocyte stage.^5^

The clinical manifestations of β-thalassemia are extremely various, spanning a broad spectrum from the transfusion-dependent state of thalassemia major to asymptomatic state of thalassemia trait. The clinical syndrome of thalassemia intermedia lies between these two clinical extremes. It comprises a wide spectrum of phenotypes from a condition that is slightly less severe than transfusion-dependent to one that is asymptomatic and often identified through a routine blood test.^2^

Soluble transferrin receptors (sTfR ) is a truncated form of tissue receptor.^6^ The bulk of sTfR measured in serum is proportional to the mass of cellular TfR and originates mostly from erythroblasts and to a lesser extent from reticulocytes.^7^ The two major determinants of s-TfR level are body iron status and the bone marrow erythroid expansion and activity. So, its level is a reliable indicator of functional iron deficiency and enhanced red cell production.^8^ It offers an advantage in assessing iron status because of its ability to distinguish hyposideremia of iron deficiency anemia (where it is elevated) from hyposideremia of inflammation anemia ( where it is not affected). Thus, it can identify the patients with inflammation and concurrent functional iron depletion, when they become iron deficient.^9^ A number of studies demonstrated that the sTfR concentration was a good indicator for evaluating the erythropoietic activity in different genotypes of thalassemia.^10^,^11^

Haptoglobin (Hp) is an abundant plasma acute phase alpha2-glycoprotein that has antioxidant and immune-modulatory properties.^12^ The plasma concentration of Hp increases several folds in the event of an inflammatory stimulus such as infection, injury or malignancy. Interleukin −6 (IL-6) is the main inducer of the expression of this protein.^13^

The primary function of Hp is to scavenge circulating hemoglobin (Hb) released by hemolysis or normal red blood cells turnover.^14^ The resulting circulating Hp-Hb complexes are rapidly eliminated from the circulation through uptake by monocytes and tissue macrophages via CD163 receptors, preventing the generation of reactive oxygen species and prevent renal damage. After endocytosis, Hp is not recycled but instead the Hp-Hb complex is degraded by lysosomes resulting in Hp depletion.^15^

Because Hp levels become depleted in the presence of large amounts of free Hb, decreased Hp is a marker of hemolysis.^16^ Plasma Hp depletion had been attributed mainly to the direct release of free Hb into the circulation during intravascular hemolysis.^17^ Even in disorders with predominantly extravascular hemolysis like thalassemia, Hb release from macrophages in the reticuloendothelial system (RES), may account for the observed Hp decrease. Further, disorders with predominantly extravascular hemolysis may gain an intravascular component, as structurally altered red cells that escaped clearance by the RES could lyse intravascularly upon prolonged circulation.^18^ As it is produced by the liver, Hp level is also decreased in hepatocellular disorders.^19^

Thus, it could be expected that the degree of Hp depletion among thalassemia patients could be affected both by the rate of hemolysis, reflecting the level of ineffective erythropoiesis, and by liver function, which could be affected by presence of Hepatitis C viral (HCV) infection and/or the iron overload. So, we investigated the relation between Hp serum level and the degree of anemia severity, positivity for HCV and iron load in the 2 thalassemia phenotypes, TM and TI.

## Materials and Methods

This is a cross sectional study that was performed upon 62 children; 42 β-thalassemia children and 20 age and sex matched healthy children who were enrolled as controls. The included children were categorized into 3 groups.

Group (1); consisted of 22 β-TM children (14 males, 8 females). Their ages ranged from 3 to 18 years with mean age of 9.9 ± 5.8 years. These patients were on a regular blood transfusion regimen (every 3–4 weeks) since infancy to maintain pre-transfusion Hb above 7 gm/dl and post transfusion Hb above 10gm/dl.

Group (2); consisted of 20 β-TI children (10 males, 10 females). Their ages ranged from 4 to 18 years with mean age of 11.8 ± 4.6 years. These patients had received only sporadic blood transfusions (less than 4 times each year).

For both patient groups, chelation therapy was usually started when serum ferritin approximated 1000 ng/ml.^20^ Chelation was either by subcutaneous Deferoxamine (DFO) infusion in a dose of 30–50 mg/kg/day, 5 days/week, by oral Deferasirox ( 20–30 mg/kg/day) or combined therapy of both DFO three days/week and daily oral Deferasirox.

Thalassemia patients were enrolled from the pediatric hematology clinic Menoufia University Hospital, Egypt.

Group (3); consisted of 20 age and sex matched healthy controls (8 males, 12 females). Their ages ranged from 3 to 18 years with mean age of 11 ± 6.9 years. They had normal complete blood count (CBC) and Hb electrophoresis with no family history of any chronic hemolytic anemia. They had been randomly selected from children presented to our general outpatient clinic for follow up, or non-specific complains.

### Exclusion criteria for included children

1. Presence of acute illness including infections.
2. Presence of diabetes mellitus or thyroid dysfunction.
3. Presence of any clinical manifestations of liver cell failure.
4. Presence of liver fibrosis or cirrhosis evident by abdominal ultrasonograhy.

The study was performed from January 2013 to August 2013. Informed consent was obtained from the legal guardians of the studied children and the ethical committee in Menoufia Medical School had approved the study.

Thalassemia children were subjected to detailed history taking and thorough clinical examination. Special emphasis was given on the age of the disease manifestation, time of the first blood transfusion, frequency of blood transfusion, with calculation of RBCs transfusion index during the last year, chelation therapy details, hepatic and renal, histories and history of splenectomy.

For all included children (patients and controls) weight and height were measured by the standard method

All included children were submitted to the following laboratory investigations:

1. Complete blood count (CBC): by using AC920 Auto-counter after calibration. Pre transfusion samples were considered for patients requiring blood transfusion at study time.
2. Markers for hepatitis B virus (HBV) and hepatitis C virus (HCV): the screening was made by enzyme-linked immunoabsorbent assay (ELISA) for hepatitis B surface antigen (HBsAg), antibody to hepatitis B core antigen (anti-HBc) and HCV antibody (HCV Ab). The positive cases were confirmed and tested for viral load by reverse transcriptase polymerase chain reaction (PCR).
3. Serum ferritin level was measured by Enzyme Linked Immune Sorbent Assay (ELISA) technique (ELISA; Ramco Laboratories Inc, Stafford, Texas, USA) on Microplate reader (Bio-Rad 680 Hercules, California, USA). The mean yearly serum ferritin level in the previous year was considered (on the average of 4 determinations) for patients and at time of sampling for the controls.

### Sample Collection and assay for other biochemical analyses

Venous blood samples were drawn by sterile vein-puncture. In patients receiving blood transfusion, samples were drawn before packed RBCs transfusion. Blood samples were immediately centrifuged for 15 minutes at 3000 rpm; sera were separated then were stored at −20°C until analysis. The serum aliquot was used for enzymatic colorimetric determination of alanine aminotransferase (ALT) and aspartate aminotransferase (AST). Serum Hp level was measured by ELISA using the Quantikine Human Haptoglobin Immunoassay (R&D Systems, Inc, Minneapolis, USA) according to the manufacturer’s protocols. Results were obtained in ng/ml and then converted to mg/dl. The minimum detectable dose (MDD) of Hp ranged from 0.031–0.529 ng/mL. The mean MDD was 0.192 ng/mL. The assay for sTfR in blood samples was performed with Human sTfR ELISA Kit (BioVendor, Research, and diagnostic products ) following the manufacturer’s protocol.

### Statistical analysis

The data were processed on an IBM-PC compatible computer using SPSS version 16 (SPSS Inc., Chicago, IL, USA). Continuous parametric variables were presented as means± SD while for categorical variables numbers (%) were used. In statistical analyses, compatibility with standard distribution was evaluated using Shapiro–Wilk normality test. Chi-square test was used for qualitative variables. The difference between 2 groups was performed by student’s t-test for parametric continuous variables and Man Whitney (U) test for non-parametric variables. Pearson correlation (r): was the test used to measure the association between two quantitative parametric variables, and Spearman correlation coefficient was applied for non-parametric data. Two-sided P-value of < 0.05 was considered statistically significant.

## Results

For TM children, their ages at diagnosis ranged from 0.5–1.5 years with a mean of 0.8± 0.25 years. The mean age of first blood transfusion was 0.7 ± 0.2 years with a range of 0.5–1 years. The mean duration of transfusion treatment was 8.9 ± 5.6 years, that of the number of the transfusions/year was 10.36 ±1.76 (median of 10 transfusions/year). While for TI children, their ages at diagnosis ranged from 3.5–7 years with a mean of 4.8± 0.9 years. The mean age of first blood transfusion was 7 ± 2.3 years with a range of 3.25–11 years. The mean duration of disease manifestations was 7 ± 4.1 years, that of the number of the transfusions/year was 2.15 ±0.75 (median of 2 transfusions/year).

The 3 groups were matched regarding age, sex, body weight and height. Hepatitis B virus infection was not found in any of the studied children. Hepatitis C virus infection was confirmed (by PCR) in 11 TM children and 8 TI children but not in any of the studied controls. All HCV infected thalassemia children had low viral load and their transaminases levels ranged between 2 to 3 folds of the average values. History of splenectomy was documented in 9 TM and 10 TI children.

**Figure 1:**
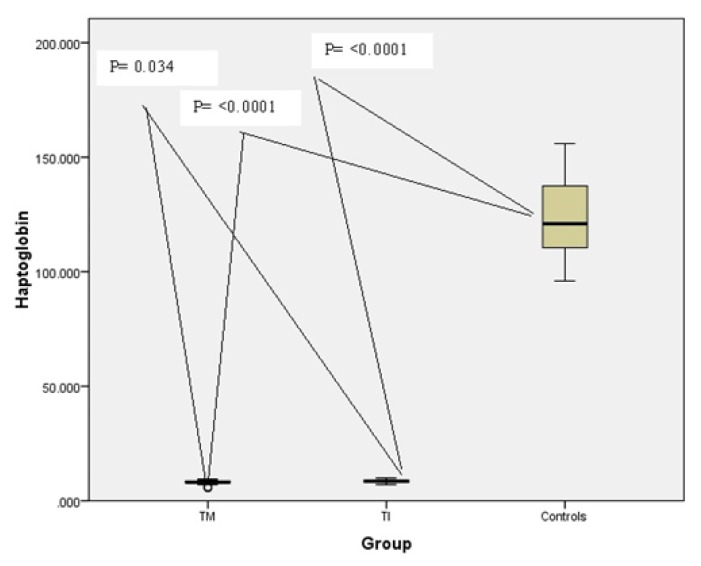
Comparison of serum Hp level (mg/dl) in TM, TI patients compared to controls.

Comparison between the studied groups regarding clinical, and laboratory data are represented in tables 1 and 2. Compared to the controls thalassemia children combined and both thalassemia groups (TM and TI) had significant lower Hb level with significantly higher ALT, AST, serum ferritin and serum TfR. Thalassemia major children had significant lower Hb level with significantly higher RBCs transfusion index and sTfR level without significant difference in any of the other tested parameters as compared to TI group. Regarding serum Hp, thalassemia children combined and both thalassemia groups had significant lower levels as compared to the control group (p<0.0001) being significantly higher in TI children (Table 1 and Figure 1).

According to the results obtained, patients were then categorized regarding positivity of HCV.

**Figure 2:**
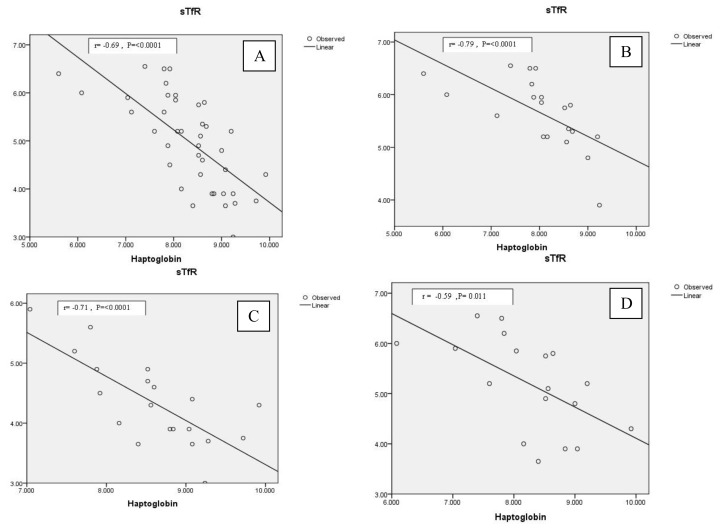
**a.**Correlation between serum Hp (mg/dl) and sTfR (μg/ml) in thalassemia children combined. **b.** Correlation between serum Hp (mg/dl) and sTfR (μg/ml) in TM children. **c.** Correlation between serum Hp (mg/dl) and sTfR (μg/ml) in TI children. **d.** Correlation between serum Hp (mg/dl) and sTfR (μg/ml) in HCV infected thalassemia children.

Thalassemia children with HCV infection had significant lower Hb with significant higher transaminases (ALT and AST) levels as compared to those free from this infection. (Table 3) Significant negative correlations were found between serum Hp and sTfR among thalassemia children combined and in each of the studied thalassemia groups (TM and TI) (Figure 2a, b and c). Not-significant correlations were found between serum Hp and the amount of RBCs transfused (RBCs TI) in thalassemia patients combined or in each of the thalassemia groups (r= −0.2, P= 0.19 for thalassemia children combined; r= −0.16, P=0.5 for TM; r= 0.19, P= 0.41 for TI )

In multivariate sTfR, concentration was the only independent predictor for serum Hp level (Table 4).

Among HCV positive patients, serum Hp level did not correlate with ALT (r=−0.19, P= 0.445), AST (r=−0.18, P= 0.46) or serum ferritin (r=0.09, P=0.71 ) but inversely related to sTfR (r= −0.59, P= 0.011) (Figure 2d). Again no significant correlation was found between serum Hp and the amount of transfused RBCs (RBCs TI ) among these patients (r = −0.23, P =0.59)

## Discussion

The plasma haptoglobin level is decreased in hemolysis as well as in presence of ineffective erythropoiesis. 19 Its depletion is a reliable marker for the instant diagnosis of accelerated red cell destruction irrespective of the site of hemolysis (intravascular or extra vascular) or the presence of concomitant inflammation.^18^ So, Hp depletion in thalassemia patients is attributed to both hemolysis and IE

In resting state, the plasma Hp levels in healthy individuals beyond the age of 4 months are measured in the 30–200 mg/dl range.^21^ Free Hp is cleared from the plasma in about 3.5–5 days, while when bound to Hb (as in the hemolytic conditions), the average time for the complex removal is about 20 minutes.^22^

To our knowledge, no data about relation between Hp and the erythropoietic activity among thalassemia children are available.

In this study, thalassemia children of both groups had severe Hp depletion as compared to the reference range and as compared to the control group. In addition, all of these children had their Hp less than 12mg/dl. In an old study, 123 patients with 16 different type of hemolytic disease (Mostly acquired, but including also 4 subjects with sickle cell disease and one with hereditary spherocytosis) all had lower plasma Hp values compared to healthy controls. The majority (81.3%) of these patients presented with Hp level of less than 12 mg/dL.^18^

It had been postulated that both hemolysis and ineffective erythropoiesis are more pronounced in TM than in TI.^2^ So, it could be expected that the degree of Hp depletion could be related to the severity of the thalassemia state. Supporting this hypothesis, the results of this work reveal that our TM children have severe anemia, while requiring greater backed cell transfusion as compared to TI children; this severity had been reflected upon their mean serum Hp level that was significantly lower than that of TI group (P = 0.034).

Circulating sTfR is proportional to erythroid precursor mass (i.e., rate of erythropoiesis).^23^

Its concentration was a good indicator for evaluating the erythropoietic activity in different genotypes of thalassemia^10^,^11^ in which the peripheral reticulocyte count is not proportional to the degree of erythropoietic activity due to the characteristic IE.^5^

Since sTFR reflects the erythropoietic activity, it was significantly higher in thalassemia patients combined, and each of TM and TI compared to the control group. Previous studies^24^,^25^ have demonstrated higher levels of serum sTfR in patients with β-thalassemia syndromes compared with the healthy controls. In accordance with this, the sTFR level was found to be significantly higher in each of TM and TI children compared to the control group. It was significantly higher in TM compared to TI patients. The significant higher sTfR in TM children compared to TI patients detected in this work, denotes that TM children have higher erythropoitic activity than TI children, finding that is discordant what was reported by Camberlein et al.^26^ who found the reverse.

This datum could be explained by considering that at variance with our series of patients, in Camberlein series the Hb was higher in TM patients than in TI patients. In fact there is a relationship between transfusion regimen and suppression of erythropoiesis in thalassemia patients that a moderate transfusion regimen may reduce iron loading in beta-thalassemia major without producing excessive expansion of erythropoiesis.^27^

Both groups of children (TM and TI) show a n inverse correlation between serum Hp as sTfR; the high sTfR level reflect the enhanced erythroid activity, which is also stimulated by anemia. Since the blood samples were taken before transfusion and absence of significant correlation between the amount of transfused backed RBCs, the depletion of Hp should be attribute primarily to intramedullary hemolysis of expanded erythroid series. In a multivariate regression analysis of some variables that could affect Hp level, sTfR was found to be the only significant predictor of serum Hp level, finding that was not valid for Hb level or RBCs TI (ml/kg/year). This means that the main features which increase sTfR level reduce also the Hp in our thalassemia children. Contemporary dosage of Hp and sTfR could be useful di find the more favorable transfusion regimen.

Haptoglobin is an acute phase protein that increases with conditions of inflammation and trauma,^14^ but decreased in hepatocellular disorders.^16^

Lifelong blood transfusion is the mainstay of thalassemia management.^1^ Transfusion-transmitted infections such as Hepatitis B Virus (HBV) and Hepatitis C Virus (HCV) are dreaded consequences of transfusions, as these can result in long-term morbidity and mortality.^28^ The frequency of HCV infection is considerably high among Egyptian children with thalassemia.^29^

In previous studies performed in populations without hemolytic disorders, low serum levels of Hp were reported in HCV infected patients with or without fibrosis compared to non-infected controls.^30^,^31^ As far as we know, there are no published data about this relation among population with thalassemia. In this study presence of HCV infection did not favor further Hp depletion as our studied thalassemia children with HCV infection had a comparable serum Hp level with those without this infection, although having lower Hb and higher serum ferritin levels. In addition, serum Hp did not show any significant correlation with liver transaminases either in thalassemia patients combined or among HCV positive cases. This is inconsistent with what was previously reported of significant negative correlation between these parameters.^31^ This difference could be explained by that our included HCV patients had low viral load and mild activity, with their transaminases levels ranged between 2 to 3 folds of the normal values. It is worth mentioning that, among these infected children, serum Hp was only inversely related to sTfR, result that further augment the association between serum Hp and the degree of ineffective erythropoiesis and eythropoietic activity. So we could speculate that the dominant effect upon serum Hp depletion was for the enhanced eythropoietic activity not for the presence of HCV infection, remembering that no one of our patients had clinical signs of liver cell failure or evidence of hepatic fibrosis or cirrhosis by ultrasonography.

Chronic iron overload is the primary cause of morbidity and mortality of thalassemia patients. It results from a number of mechanisms including repetitive blood transfusion, peripheral hemolysis, increased intestinal iron absorption as well as ineffective erythropoiesis.^4^

Serum ferritin is one of the acute phase proteins that could increase in conditions of infections and inflammations.^32^ Although it was shown to be a poor predictor of iron load as it is affected by other conditions, serum ferritin level is still the widely and most commonly used method to asses and monitor iron load in thalassemia children.^33^ As expected, in this study the mean yearly serum ferritin was significantly higher in both thalassemia groups (TM and TI) when compared to the control group, without significant difference between them. In this study, serum ferritin was significantly higher in HCV positive thalassemia children compared to HCV negative patients. This is consistent with what was previously reported by Atta et al.^31^

Investigating the relation between serum Hp as a hemolytic marker and the mean yearly serum ferritin as a marker of iron overload in thalassemia children, we did not detect significant correlation between them either in all studied thalassemia children or among the individual groups (TM and TI) each separately. This means that the degree of serum Hp depletion although is related to the degree of erythropoitic activity, it is not influenced by iron overload. Indeed, iron load in thalassemia is a complex process that is affected by the iron chelation type as well as its compliance.^34^

In summary, Hp depletion is a reliable marker for the instant diagnosis of accelerated red cell destruction irrespective of the site of hemolysis or the presence of inflammation.^18^

In this study serum Hp level was more depleted in TM than in TI children and had significant inverse correlation to the degree of eythropoietic activity assessed by sTfR which was the only predictor of its level. Also this level did not show any difference regarding the presence of HCV infection, and did not correlate to liver transaminases.

Indeed, Hp concentration is not only determined by hemolysis and the acute phase response but also by its phenotype.^35^ It is known that Hp exists in three phenotypic forms, Hp1-1, 2-1, and 2-2, encoded by two co-dominant alleles, Hp1 and Hp2.^36^ The ability to bind Hb is phenotype-dependent and has been found to be in the order 1-1 > 2-1 > 2-2. The binding capacity reflects the plasma Hp levels of the three phenotype being highest in Hp 1-1 and lowest in Hp 2-2 phenotypes.^37^ So, our results raise the concern about the importance of investigating Hp phenotyping in thalassemia patient to find out its possible impact on Hp level among thalassemia patients.

## Conclusions

Serum Hp level was had good relation to hemolysis severity among thalassemia children and could be predictive of the degree of ineffective erythropoiesis without significant relation to HCV infection and did not reflected on enhanced iron overload. Large sample multicenter studies and Hp phenotyping for different thalassemia categories are highly recommended.

## Figures and Tables

**Table 1:** Comparison between the patients and the controls regarding demographic, clinical, and laboratory data

Variables	Patients (42)	Control (20)	P value
Age (years)	10.87± 5.28	11 ± 6.9	0.97
Sex, male, n(%)	24 (57.1)	8(40)	0.21
Consanguinity	17 (40.5)	3(15)	**0.045**
Body weight (Kg )	30.98 ± 14.62	35.1 ±23.1	0.97
Height (cm)	133.64±23.26	132.7±33.8	0.84
Chelation, n (%)			
• DFO	19 (45.2)		
• Deferazirox	21 (50)	-	-
• Combined	2 (4.8)		
HCV infection	19 (45.2)	-	-
Splenectomy, n (%)	19 (45.2)	-	-
RBCs TI (ml/kg/year)	75.97 ± 35.98	-	-
Hb level (g/dl)	7.15 ± 1.23	11.7 ± 0.8	**<0.0001**
ALT (IU/L)	56.57 ± 48.65	19.2 ± 3.5	**<0.0001**
AST (IU/L)	59.35 ± 46.88	17.6 ± 3.4	**<0.0001**
Serum ferritin(ng/ml)	3313.7 ± 2723.06	99.5 ± 15.9	**<0.0001**
Serum Hp (mg/dl)	8.31 ± 0.87	122 ± 18.5	**<0.0001**
Serum TfR (μg/ml)	4.99 ± 0.95	0.88±0.69	**<0.0001**

Bold numerical values indicate significance.

**Table 2:** Comparison between the studied groups regarding demographic, clinical, and laboratory data.

Variables	TM (22)	TI (20)	Control (20)	P1	P2	P3
Age (years)	9.9 ± 5.8	11.8 ± 4.6	11 ± 6.9	0.7	0.77	0.25
Sex, male, n(%)	14 (63.6)	10 (50%)	8(40)	0.13	0.52	0.37
Consanguinity	12(54.5)	5 (25)	3(15)	0.008	0.25	0.051
Body weight (Kg )	27 ± 12.2	34.9 ± 15.9	35.1 ±23.1	0.66	0.71	0.80
Height (cm)	127.9±25.2	139.3±20.2	132.7±33.8	0.43	0.65	0.12
Chelation, n (%)						
• DFO	12 (54.5)	7 (35)				
• Deferazirox	9 (41)	12(60)	-	-	-	0.44
• Combined	1 (4.5)	1(5)				
HCV infection	11 (50)	8(40)	-	-	-	0.52
Splenectomy, n(%)	9 (41)	10(50)	-	-	-	0.55
RBCs TI (ml/kg/year)	98.7 ± 31.7	53.2 ± 23.7	-	-	-	**<0.0001**
Hb level (g/dl)	6.5 ± 0.9	7.7 ± 1.2	11.7 ± 0.8	**<0.0001**	**<0.0001**	**0.001**
ALT (IU/L)	64.1 ± 61.3	49 ± 31.3	19.2 ± 3.5	**0.003**	**<0.0001**	0.92
AST (IU/L)	66.1 ± 53.8	52.6 ± 38.9	17.6 ± 3.4	**<0.0001**	**<0.0001**	0.37
Serum ferritin(ng/ml)	3417 ± 2988	3210 ± 2500	99.5 ± 15.9	**<0.0001**	**<0.0001**	0.8
Serum Hp (mg/dl)	8.02 ± 0.93	8.6 ±0.72	122 ± 18.5	**<0.0001**	**<0.0001**	**0.034**
Serum TfR (μg/ml)	5.65 ± 0.66	4.34 ± 0.71	0.88±0.69	**<0.0001**	**<0.0001**	**<0.0001**

P1 for comparison between TM and controls.

P2 for comparison between TI and controls.

P3 for comparison between TM and TI.

Bold numerical values indicate significance.

**Table 3:** Comparison of demographic, clinical and laboratory data between HCV positive and HCV negative thalassemia children.

Variables	Hepatitis positive (19/42)	Hepatitis negative (23/42)	P Value
Age (years)	14 ± 4.9	8.2± 4	**<0.0001**
Male; n (%)	12(66.7)	11 (50)	0.289
Body weight	39 ± 14.5	24.3 ± 11.1	**0.001**
RBCs TI (ml/kg/year)	65.4 ± 37.8	84.5 ± 32.8	0.094
Hb level (g/dl)	6.7 ± 0.66	7.5 ± 1.4	**0.035**
ALT (IU/L)	58.8 ± 56	32.6 ± 22.8	**<0.0001**
AST (IU/L)	92.5 ± 46.8	32.1 ± 24	**<0.0001**
Blood urea(mg/dl)	15.4 ± 3.8	17.3 ± 4.1	0.14
Serum creatinine (mg/dl)	0.47 ± 0.14	0.51 ± 0.14	0.38
Serum ferritin(ng/ml)	4272 ± 2643	2529 ± 2585	**0.042**
Serum Hp (mg/dl)	8.26 ± 0.89	8.35 ± 0.88	0.73
Serum TfR (μg/ml)	5.19 ± 0.94	4.83 ± 0.96	0.24

Bold numerical values indicate significance.

**Table 4:** Multiple linear regression analysis for independent variables affecting the serum Hp level in β-thalassemic children.

	Un-standardized Coefficient		Standardized Coefficient	t	P

	B	SE	Beta		
Constant	11.937	1.262		9.462	< 0.0001
Age (years)	0.027	0.034	0.161	0.792	0.434
Hb (g/dl)	−0.082	0.100	−0.115	−0.818	0.419
Amount of transfused RBCs (ml/kg/yr)	0.004	0.006	0.169	0.741	0.463
sTFR (μg/ml)	−0.729	0.130	−0.797	−5.585	**< 0.0001**
Dependent Variable: Hp (mg/dl)					

Bold numerical values indicate significance.
